# “A cleaner break”: Genetic divergence between geographic groups and sympatric phenotypes revealed in ballan wrasse (*Labrus bergylta*)

**DOI:** 10.1002/ece3.6404

**Published:** 2020-06-05

**Authors:** Gaute W. Seljestad, María Quintela, Ellika Faust, Kim T. Halvorsen, François Besnier, Eeva Jansson, Geir Dahle, Halvor Knutsen, Carl André, Arild Folkvord, Kevin A. Glover

**Affiliations:** ^1^ Institute of Marine Research Bergen Norway; ^2^ Department of Biological Sciences University of Bergen Bergen Norway; ^3^ Department of Marine Sciences—Tjärnö University of Gothenburg Strömstad Sweden; ^4^ Institute of Marine Research Austevoll Research Station Storebø Norway; ^5^ Institute of Marine Research Flødevigen Norway; ^6^ Centre for Coastal Research University of Agder Kristiansand Norway

**Keywords:** aquaculture, cleaner fish, fisheries management, microsatellite, SNP, translocation

## Abstract

Capture and long‐distance translocation of cleaner fish to control lice infestations on marine salmonid farms has the potential to influence wild populations via overexploitation in source regions, and introgression in recipient regions. Knowledge of population genetic structure is therefore required. We studied the genetic structure of ballan wrasse, a phenotypically diverse and extensively used cleaner fish, from 18 locations in Norway and Sweden, and from Galicia, Spain, using 82 SNP markers. We detected two very distinct genetic groups in Scandinavia, northwestern and southeastern. These groups were split by a stretch of sandy beaches in southwest Norway, representing a habitat discontinuity for this rocky shore associated benthic egg‐laying species. Wrasse from Galicia were highly differentiated from all Scandinavian locations, but more similar to northwestern than southeastern locations. Distinct genetic differences were observed between sympatric spotty and plain phenotypes in Galicia, but not in Scandinavia. The mechanisms underlying the geographic patterns between phenotypes are discussed, but not identified. We conclude that extensive aquaculture‐mediated translocation of ballan wrasse from Sweden and southern Norway to western and middle Norway has the potential to mix genetically distinct populations. These results question the sustainability of the current cleaner fish practice.

## Graphical abstract

The genetics of ballan wrasse, a phenotypically diverse and extensively used cleaner fish, was studied along the Scandinavian coastline and in Galicia, Spain. A distinct genetic break was detected in Scandinavia, dividing northwestern and southeastern clusters. Additionally, distinct genetic differences were observed between sympatric spotted and plain phenotypes in Galicia, but not in Scandinavia.

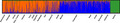

## 1 INTRODUCTION

Unlike capture‐based fisheries, global aquaculture production has increased markedly over the past decades and has major prospects of further expansion (FAO, 2018). As such, it is often viewed as one of the most important sources of food for the growing human population. However, depending on the nature, location, volume of production, and farmed species in question, aquaculture may elicit a variety of negative effects on wild populations and the surrounding environment. For salmonid aquaculture that is conducted in open sea‐cages in both the Atlantic and Pacific oceans, sea lice, which infest both farmed and wild salmonids, represent one of the most significant and persistent challenges (Taranger et al., 2015; Torrissen et al., 2013).

While a diverse set of sea lice control strategies has been implemented by the industry, the use of cleaner fish that eat lice from farmed salmonids in sea‐cages has been presented as a sustainable and ecologically sound approach. Cleaner fish were introduced in salmonid aquaculture in the late 1980s (Bjordal, 1988; Darwall, Costello, Donnelly, & Lysaght, 1992; Rae, 2002) although the use dwindled in the late 1990s and mid‐2000s due to widespread chemotherapeutant use. However, as lice rapidly developed a high level of resistance (e.g., Espedal, Glover, Horsberg, & Nilsen, 2013; Fallang, Denholm, Horsberg, & Williamson, 2005; Fallang et al., 2004; Kaur et al., 2017; Ljungfeldt, Espedal, Nilsen, Skern‐Mauritzen, & Glover, 2014; Treasurer, Wadsworth, & Grant, 2000), cleaner fish use has since had a resurgence and currently exceeds 48 million fish per year in Norway alone (Norwegian Directorate of Fisheries, 2019). The primary cleaner fish species currently in use include lumpfish (*Cyclopterus lumpus* L., 1758) and four species of wrasse: goldsinny (*Ctenolabrus rupestris* L., 1758), corkwing (*Symphodus melops* L., 1758), rock cook (*Centrolabrus exoletus* L., 1758), and ballan (*Labrus bergylta* Ascanius, 1767) (Blanco Gonzalez & de Boer, 2017; Darwall et al., 1992; Powell et al., 2017; Skiftesvik et al., 2014).

Of the species used as cleaner fish, ballan wrasse is the most valuable due to its hardiness and large size (Blanco Gonzalez & de Boer, 2017; Skiftesvik, Bjelland, Durif, Johansen, & Browman, 2013). It is a hard‐bottom associated species, with a natural distribution from the Iberian Peninsula to a northern terminus in Trøndelag, mid‐Norway (Quignard & Pras, 1986). Reaching an age of more than 25 years (Dipper, Bridges, & Menz, 1977; Dipper & Pullin, 1979) and a size of up to 60 cm (Quignard & Pras, 1986), it is the largest and longest living of the wrasse in northern Europe. Ballan wrasse is a monandric protogynous hermaphrodite; that is, all fish are born female and change sex to become males at ~6 years of age, usually before reaching 40 cm in length (Darwall et al., 1992; Dipper & Pullin, 1979; Muncaster, Norberg, & Andersson, 2013). Protogynous life histories tend to result in highly female skewed population sex ratios (Allsop & West, 2004), leading to smaller effective population size (*N*_e_), which in turn increases the likelihood of developing population structure (D'Arcy, 2013). In addition, ballan wrasse show strong site affiliation and have small home ranges (Mucientes, Irisarri, & Villegas‐Ríos, 2019; Skiftesvik, Durif, Bjelland, & Browman, 2015; Villegas‐Ríos, Alós, et al., 2013). Males also keep harems of females with which they spawn on the benthic substrate (Darwall et al., 1992). The combination of these traits is thus likely to generate population structure (Dipper et al., 1977; Dipper & Pullin, 1979).

Ballan wrasse are phenotypically diverse and display two main phenotypes, spotty and plain, which can be found in sympatry. In Galicia, northwest Spain, these two phenotypes have different common names and display differing life histories, with spotty fish growing larger and faster than plain fish that invest in earlier reproduction (Villegas‐Ríos, Alonso‐Fernández, Domínguez‐Petit, Alonso‐Fernández, Domínguez‐Petit, & Saborido‐Rey, 2013). A genetic difference between these two phenotypes in Galicia has been revealed through microsatellite DNA analysis (Quintela et al., 2016), and assortative mating has been proposed as a mechanism to maintaining the difference (Villegas‐Ríos, Alonso‐Fernández, Domínguez‐Petit, et al., 2013). These two phenotypes can also be found in other parts of the distribution range (Villegas‐Ríos, Alonso‐Fernández, Fabeiro, Alonso‐Fernández, Fabeiro, Bañón, & Saborido‐Rey, 2013), but the genetics underlying the phenotypic divergence is yet to be examined in these areas.

The majority of wrasses used as cleaner fish in aquaculture are wild captured. Currently, the Norwegian labrid fishery is divided into three management regions, where the allocation of quotas, fishing‐gear regulations, and closed seasons differs. However, knowledge of population genetic structure was not available when these management areas were initially decided upon. Of the total 18 million wrasse quota, 4 million are allocated to the southernmost management region where there are virtually no salmonid farms. From this area, wrasses are transported by trucks or boats to salmonid farms in western and middle Norway. In addition, approximately 1 million wrasses are imported annually from Sweden, and permits have also been granted for imports from Denmark although a commercial fishery is yet to be established in the latter region (Rueness et al., 2019).

Little is still known about the fate of cleaner fish in sea‐cages, but escapes and hybridization with wild populations have been documented (Faust, Halvorsen, Andersen, Knutsen, & André, 2018). Furthermore, deliberate release of wrasses following the end of salmonid production has also been reported (Taranger, Svåsand, Kvamme, Kristiansen, & Boxaspen, 2014). Thus, this practice can lead to gene flow between previously isolated populations, as suggested for goldsinny (Jansson et al., 2017) and reported for corkwing wrasse (Faust et al., 2018). This type of human‐mediated movement of organisms between areas, termed translocation, has been identified as an increasing threat to biodiversity and genetic diversity worldwide (Laikre, Schwartz, Waples, & Ryman, 2010; Prenter, MacNeil, Dick, & Dunn, 2004), through co‐introduction of (novel) disease vectors and/or parasites and by leading to extrinsic outbreeding depression and/or breakdown of population structure (André et al., 2011; Glover et al., 2017).

The population genetic structure of ballan wrasse has yet to be studied in Scandinavia. Given the widespread harvest and translocation of this species, such studies are required to provide sound management advice. Thus, the present study was designed with two primary objectives: (a) to investigate the population genetic structure of ballan wrasse in Scandinavia, including comparative analysis with Galician populations, and (b) to investigate the relationship between genetic and phenotypic variation in this species in both Scandinavia and Galicia. In order to achieve these objectives, we genotyped 1,058 ballan wrasse from Scandinavia and Galicia in Spain, using a newly developed SNP panel and existing microsatellite markers.

## 2 MATERIALS AND METHODS

### 2.1 Sampling

**FIGURE 1:**
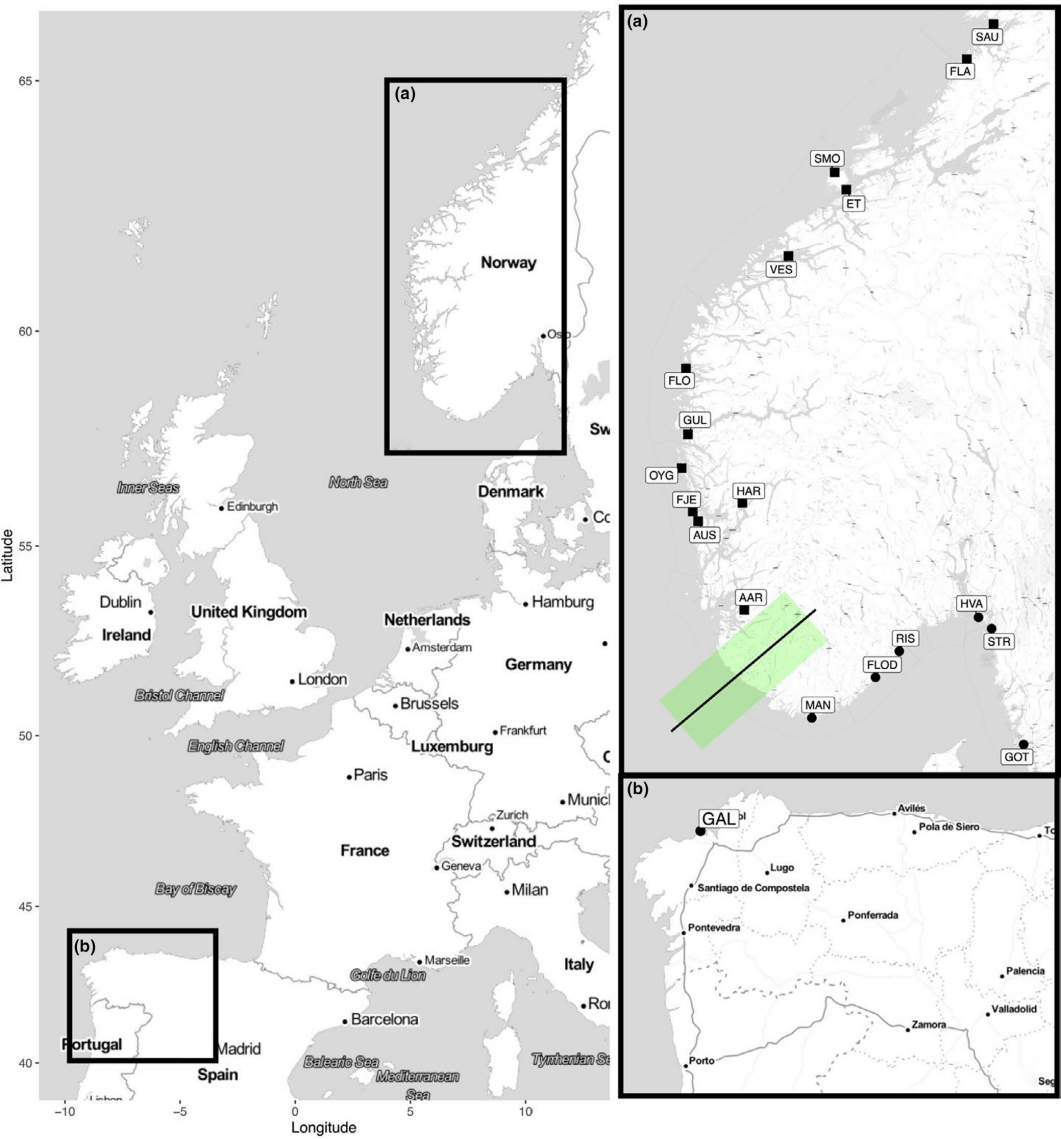
Map of the study area of ballan wrasse (*Labrus bergylta*). (a) Scandinavian sampling locations. Northwestern Scandinavian locations indicated by squares, southeastern by circles. Approximate genetic cline center and range in Scandinavia indicated by line and area shaded in green. (b) Galician sampling location. See Table 1 for detailed information about the sampling locations

**FIGURE 2:**
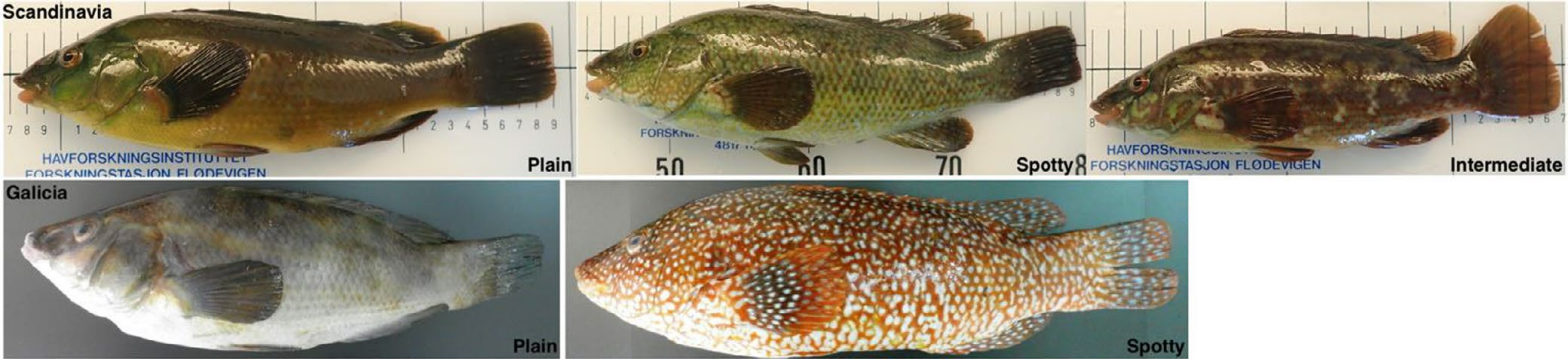
Example photographs of ballan wrasse phenotypes. The upper row shows examples of Scandinavian plain spotted and intermediate fish (caught in Flødevigen, Norway), while the lower row shows plain and spotted fish from Galicia, Spain

A total of 1,058 ballan wrasse were sampled from 16 locations in Norway, two in Sweden and one in Galicia, Spain (Figure 1). At five locations (see Table 1), fish were photographed for phenotypic analysis of plain and spotty morphs. These individuals were visually sorted according to phenotype (see example in Figure 2) in order to investigate the potential connection between phenotype and genetic background and were kept separate for analyses where appropriate.

**TABLE 1:** Summary statistics of sampled ballan wrasse (*Labrus bergylta*) using 82 single nucleotide polymorphisms (SNPs). Lat and Lon are latitude and longitude of the sampling location, respectively; *N* is the number of individuals sampled; HWE and LD are number of deviations from Hardy–Weinberg equilibrium and linkage equilibrium, respectively; *H*_e_ and *H*_o_ are expected and observed heterozygosity, respectively; and *F*_IS_ is the inbreeding coefficient. Numbers in parentheses (B) are after sequential Bonferroni corrections. Galician sample listed both as total sample and as sorted by morphotype (Plain/Spotted)

Area	Sampling location	Short code	Lat	Lon	Year(s)	*N*	Photographs	HWE (B)	LD (B)	*H*_e_	*H*_o_	*F*_IS_
NW	Sauhestøya	Sau	64.78	11.21	2017	33		6 (1)	183 (1)	0.414	0.416	0.019
	Flatanger	Fla	64.47	10.65	2017, 2018	57		9 (1)	168 (0)	0.410	0.401	0.040
	Smøla	Smo	63.45	7.93	2017, 2018	36	Y	5 (0)	162 (0)	0.413	0.407	0.025
	Edøy‐Tustna	ET	63.29	8.17	2017	25		5 (0)	136 (0)	0.403	0.399	0.033
	Vestnes	Ves	62.67	6.98	2017	81		8 (1)	152 (0)	0.409	0.411	0.007
	Florø	Flo	61.59	4.87	2018	29		6 (0)	143 (0)	0.412	0.414	0.010
	Gulen	Gul	60.94	4.91	2018	29		6 (0)	145 (0)	0.406	0.413	0.006
	Øygarden	Oyg	60.60	4.78	2018	23		7 (0)	130 (0)	0.393	0.414	−0.027
	Hardanger	Har	60.25	6.03	2012–2013	50		6 (0)	150 (0)	0.403	0.411	−0.012
	Fjell	Fje	60.16	5.01	2018	26		6 (0)	124 (0)	0.397	0.399	0.023
	Austevoll	Aus	60.06	5.12	2013	89		11 (0)	153 (0)	0.409	0.414	−0.007
	Årdal	Aar	59.14	6.07	2018	22		4 (0)	150 (0)	0.404	0.426	−0.025
SE	Mandal	Man	57.98	7.46	2018	50		6 (0)	163 (1)	0.398	0.427	−0.056
	Flødevigen	Flod	58.42	8.77	2013, 2018	197	Y	7 (4)	144 (0)	0.398	0.393	0.022
	Risør	Ris	58.70	9.26	2018	50		5 (1)	157 (1)	0.391	0.402	−0.018
	Hvaler	Hva	59.06	10.89	2012	50		6 (0)	146 (0)	0.390	0.375	0.040
	Strömstad, Sweden	Str	58.94	11.16	2018	47	Y	7 (0)	163 (0)	0.395	0.389	0.025
	Gothenburg, Sweden	Got	57.69	11.82	2018	49	Y	6 (0)	162 (1)	0.395	0.390	0.026
SP	Galicia, Spain	Gal	43.39	−8.43	2014	82	Y	9 (2)	281 (4)	0.412	0.385	0.067
	Galicia Plain	GalP	43.39	−8.43	2014	43		8 (2)	287 (0)	0.410	0.396	0.049
	Galicia Spotted	GalS	43.39	−8.43	2014	39		4 (2)	136 (0)	0.391	0.373	0.054

Scandinavian fish were caught using fyke nets or pots. Fish were killed and thereafter sampled by trained individuals in accordance with EU Directive 2010/63/EU and national legislations. Galician samples were obtained from a local fish market in A Coruña (43.359 N, 8.402 W). All fin clips were stored in 96% ethanol for later analysis.

### 2.2 Identification of single nucleotide polymorphism (SNP) markers for genotyping

Putative SNPs were randomly identified from polymorphisms identified in the genome by mining RAD sequences from ballan wrasse available from NCBI (BioSample: SAMEA2707596; SRA: ERS529359) and aligned to the genome assembly available from NCBI (BioSample: SAMEA3939555, BioProject: PRJEB13687, https://www.ncbi.nlm.nih.gov/assembly/GCF_900080235.1/) using BWA (Li & Durbin, 2009). SNPs were retrieved from the aligned sequences by using Samtools mpileup (Li, 2010, 2011; Li et al., 2009) and filtered using bcftools (Danecek et al., 2011) in order to keep the variants with high confidence call (bcftools filter ‐e “%QUAL < 100”).

Putative SNPs, identified from the procedure above, were imported in R v.3.5.0 (R Core Team, 2018) and filtered to only retain SNPs with the highest confidence of polymorphism based on the Phred‐scaled genotype likelihoods of the.vcf output file from bcftools. To cover as much as possible of the genome, as well as to avoid strong linkage between markers, one SNP was randomly selected from each of the 120 largest contigs of the assembly. A total of 106 of these identified SNPs were then sorted into four genotyping multiplexes, each consisting of 24–29 SNPs named BaWr‐1 to −106 (see Tables S1–S4 for multiplex designs).

### 2.3 Genotyping

Total genomic DNA was extracted from fin clips using the Qiagen DNeasy 96 Blood & Tissue Kit following the manufacturer's instructions (Qiagen, 2016). All samples were genotyped for the 106 SNPs on a MassARRAY^®^ platform using an iPLEX^®^ reaction (Agena Bioscience, n.d.). SNPs were amplified in 5 ml of PCR master mix consisting of 0.5 μM Multiplex primer mix (one of four multiplexes), 25 mM dNTP, 25 mM MgCl_2_, PCR buffer, 5 U/μl PCR enzyme, ~15 ng/μl DNA, and HPLC‐grade water. Cycling conditions were 2‐min denaturing at 95°C followed by 45 cycles of 30 s at 95°C, 30 s at 56°C, and 1 min at 72°C, and finished with 5 min at 72°C. After initial PCR, the remaining primers and nucleotides were removed using Shrimp alkaline phosphatase (SAP) and followed by iPLEX (Agena Bioscience™) Extension Reaction, with extension primer (UEP) mix according to multiplex (see Tables S1–S4 in Appendix S1). SAP cycling conditions were 40 min at 37°C, followed by 5 min at 85°C, while iPLEX cycling conditions were 30 s at 94°C, followed by 40 cycles consisting of 5 s at 94°C and 5 cycles of 5 s at 52°C and 5 s at 80°C. The iPLEX extension reaction was finished off by 3 min at 72°C. All thermocycler programs were set infinitely to 4°C after the cycling program was run to prevent product degradation.

Extension products were desalted using Clear Resin before transfer onto a SpectroCHIP Array by automated nanodispenser (Agena Bioscience™ RS1000). SpectroCHIP arrays were analyzed using a MALDI‐TOF Mass Spectrometer (Agena Bioscience™ MassARRAY Dx Analyzer) that assigns genotypes to sample fragments according to mass. The results were called and control checked in TyperAnalyzer v.4.1.83 (Agena Bioscience™), and all genotypes with a mass height below 0.4 were filtered.

In addition to genotyping the entire dataset with SNPs as described above, a subset of the samples (*N* = 513) were also genotyped for nineteen microsatellite loci (WrA103, WrA107, WrA111, WrA112, WrA113, WrA203, WrA223, WrA224, WrA236, WrA237, WrA254, WrA255, WrA256, WrA259, WrA261, WrB102, WrB212, WrB213, and WrB215; Quintela et al. (2014)) Microsatellites were genotyped to compare the amount of genetic diversity in samples from the southern and northern range of the species distribution. Microsatellites (six multiplexes) were amplified in 10 μl of PCR master mix, consisting of 50 ng DNA template, 1× buffer, 2 mM MgCl_2_, 1.25 mM dNTPs, 0.06–0.12 μM of each primer, and 1 U Go‐Taq polymerase. Cycling conditions were as follows: 4‐min denaturation at 94°C followed by 24 cycles of 50 s at 94°C, 90 s at an annealing temperature of 56°C, 1 min of extension at 72°C, and a final extension of 72°C for 10 min. Forward primers were labeled with fluorescent dyes and PCR products were electrophoresed on an ABI Prism 377 Genetic Analyzer (Applied Biosystems). The 500LIZ size standard (Applied Biosystems) was used to accurately determine the size of the fragments and allelic variation. Fragments were analyzed with the software GeneMapper v5 (Applied Biosystems).

### 2.4 Data analysis

Prior to any statistical analysis, extensive data quality checks were performed on the SNPs to ensure good clustering and low levels of missing data (<30%). Twenty‐four of the SNPs did not pass the quality checks, leaving the final dataset to consist of 82 SNPs. Individuals missing ≥50% of genotype calls were excluded from the analysis.

Summary statistics, sample size, observed and expected heterozygosity, as well as inbreeding coefficient (*F*_IS_) were computed using the packages *adegenet* and *hierfstat* (Goudet, 2005; Jombart, 2008) as implemented in R. Hardy–Weinberg equilibrium (HWE) tests were conducted for each sample and locus combination using the package *pegas* (Paradis, 2010) in R, using 1,000 Monte Carlo permutations. Statistical significance was assessed after post hoc sequential Bonferroni correction (Holm, 1979). Potential linkage disequilibrium (LD) between all locus pairs per population as well as across all populations was examined using the program GENEPOP 7 (Rousset, 2008) with 10,000 steps of dememorization, 100 batches, and 5,000 iterations per batch. As with HWE, significance was assessed after Bonferroni correction. All population structure analyses were conducted with the SNPs, while the microsatellite dataset was only used for genetic diversity analyses by calculating the number of alleles and allelic richness per sample using the *hierfstat* R package.

The following analyses were conducted with the SNP dataset only. To test if loci deviated from neutrality, outlier analyses were conducted using LOSITAN (Antao, Lopes, Lopes, Beja‐Pereira, & Luikart, 2008), BayeScan (v.2.1; Foll and Gaggiotti (2008)), and PCAdapt (Luu, Bazin, & Blum, 2017). Following a consensus approach, only those loci marked as under selection by at least two programs were treated as outliers. BayeScan was run by setting sample size to 10,000 and the thinning interval to 50 as suggested by Foll and Gaggiotti (2008). Loci with a posterior probability over 0.99 were retained as outliers, corresponding to a Bayes factor > 2 (i.e., “decisive selection” (Foll & Gaggiotti, 2006)). In LOSITAN, a neutral distribution of *F*_ST_ with 100,000 iterations was simulated, with forced mean *F*_ST_ at a significance level of .05 under an infinite alleles model. The third approach was based on the allele frequency method implemented in PCAdapt, in which population structure is first assessed via PCA, and outliers are thereafter detected with respect to how they relate to population structure. Cattell's scree test (Cattell, 1966) was used to select the number of principal components (K) to identify SNPs deviating from neutrality.

Bayesian clustering analysis was carried out in STRUCTURE v.2.3.4 (Pritchard, Stephens, & Donnelly, 2000) using an admixture model and correlated allele frequency without population information. The analysis was conducted using the program ParallelStructure which speeds up analytical time by dispersing runs onto multiple processors in tandem (Besnier & Glover, 2013). Ten runs with a burn‐in of 100,000 and 1,000,000 Markov chain Monte Carlo (MCMC) iterations were performed for *K* = 1–5. To determine the most likely number of genetic clusters within the data, STRUCTURE output was analyzed using two approaches. The ad hoc summary statistic Δ*K* and mean LnP(*K*) of Evanno, Regnaut, and Goudet (2005) were calculated; in addition, four alternative statistics (MedMed, MedMean, MaxMed, and MaxMean) were estimated using StructureSelector (Li & Liu, 2018). Finally, the ten iterations of the selected Ks were averaged with CLUMPP v.1.1.1 (Jakobsson & Rosenberg, 2007) using the FullSearch algorithm and the G' pairwise matrix similarity statistic and graphically displayed using bar plots. Genetic clustering was also visualized using discriminant analysis of principal components (DAPC; Jombart, Devillard, and Balloux (2010)) in *adegenet*, with grouping based on sampling locations. The number of principal components kept was determined from the number of PCAs needed to explain more than 80% of the variation. Genetic structure was assessed both using the total suite of SNP markers and using only the putative neutral loci (Figures S6 and S7). Additionally, STRUCTURE was also run separately for each location with phenotype sorted samples at *K* = 2. Pairwise *F*_ST_ matrices (Nei, 1987) were calculated in Arlequin v.3.5.1.2 (significance estimated after 10,000 permutations; Excoffier, Laval, & Schneider, 2005; Excoffier & Lischer, 2010).

A geographic cline analysis was conducted of the Scandinavian samples using the R package *HZAR* (Derryberry, Derryberry, Maley, & Brumfield, 2014) over a 1,500‐km transect starting in Sauhestøya, Norway, and finishing in Gothenburg, Sweden (i.e., from 64.783 N to 57.691 N; Figure 1). The fifteen models implemented in *HZAR* were fitted to the normalized loading on the first principal component analysis (PCA) axis both based on the panel of 82 SNPs and to the allele frequency of every individual locus to determine the position, width, and shape of clines over the total geographic distance. The reference cline was built using STRUCTURE Q‐score for the total dataset and, in both cases, the best cline model was decided upon AIC scores. Clines were considered significantly displaced if the two log‐likelihood unit support limits of the cline center did not overlap with the STRUCTURE Q‐score (Qb = 1 − Qs).

Isolation‐by‐distance (IBD) analysis was computed for the entire Scandinavian dataset as well as within the clusters detected by pairwise *F*_ST_ and STRUCTURE conducted using the R packages *adegenet* and *ade4* (Dray & Dufour, 2007). Nei's genetic distance was calculated by adegenet, while geographic distance was approximated by following the coastline in Google Maps.

## 3 RESULTS

The final dataset consisted of 1,025 individuals from 19 locations genotyped for 82 SNPs, and a subset of 513 individuals from 8 locations genotyped for 19 microsatellites. In addition, 325 individuals from five locations were also phenotype sorted (spotty and plain). It is important to note that microsatellites were only genotyped to provide supplementary information on genetic variation in some of the samples, that is, number of alleles and allelic richness.

Out of the 82 SNP loci, five deviated from HWE, and eight displayed pairwise LD. Only one locus deviated in these parameters in more than one sampling location; that is, BaWr40 departed from HWE in Flødevigen and Flatanger, and thus, all loci were kept in all subsequent analyses. Overall observed heterozygosity was similar across sampling locations for SNPs (Table 1), with values ranging from 0.375 to 0.426 in Scandinavia, and 0.385 for the Galician sample.

For microsatellites (Table 2), the total number of alleles (*N*_A_) and allelic richness (*A*_R_) per sample in Scandinavia ranged from 69 to 99 (mean = 84) and 3.8 to 4.0 (mean = 4.0), respectively. In Galicia, values were considerably higher (*N*_A_ = 139, *A*_R_ = 5.9), thus showing greater genetic diversity in southern latitudes. This was also reflected in the higher estimate of heterozygosity for the microsatellites in the Galician sample (0.620) versus the values in Scandinavia (0.493–0.540, mean 0.516).

**TABLE 2:** Summary statistics for the microsatellite data subset: Number of sampled individuals (*N*), number of alleles (*N*_A_) per sampling location, allelic richness (*A*_R_), number of loci deviating from Hardy–Weinberg expectations (HWE) and locus pairs in linkage disequilibrium (LD), expected and observed heterozygosity (*H*_e_, *H*_o_), and inbreeding coefficient (*F*_IS_). Numbers in parentheses after sequential Bonferroni corrections (B). Galician sample listed both as total sample and as sorted by phenotype (Plain/Spotted)

Area	Sampling location	*N*	*N*_A_	*A*_R_	HWE (B)	LD (B)	*H*_e_	*H*_o_	*F*_IS_
NW	Vestnes	85	99	4.3	3 (2)	11 (0)	0.566	0.540	0.059
	Hardanger	38	72	3.8	2 (0)	5 (0)	0.567	0.525	−0.016
	Austevoll	88	94	4.0	2 (1)	10 (0)	0.544	0.536	0.023
SE	Flødevigen	102	92	3.9	3 (3)	9 (0)	0.513	0.512	−0.001
	Hvaler	22	69	3.8	1 (1)	6 (0)	0.538	0.493	−0.004
	Strömstad	44	81	3.9	1 (1)	9 (0)	0.515	0.497	0.047
	Gothenburg	50	84	4.0	3 (1)	10 (0)	0.521	0.508	0.035
SP	Galicia, Spain	89	139	5.9	3 (1)	17 (1)	0.629	0.620	0.023
	Galicia Plain	48	127	5.9	1 (1)	17 (0)	0.638	0.640	0.002
	Galicia Spotted	41	121	5.7	2 (0)	10 (1)	0.600	0.600	0.022

### 3.1 Outlier tests

The three approaches implemented to detect outlier SNPs produced three consensus loci (BaWr‐46, BaWr‐60, and BaWr‐82) across the 18 locations in Scandinavia (between northwestern [NW] and southeastern [SE]) and four between the phenotypic groups in Galicia (BaWr‐22, BaWr‐32, BaWr‐58, and BaWr‐97). Pairwise *F*_ST_ analysis, STRUCTURE analysis, and DAPC were conducted both with and without outliers and because only marginal differences in the results were observed between the two sets of loci (Figure S2), all markers were retained for subsequent analyses.

### 3.2 Genetic differentiation and allele frequency patterns

Genetic differentiation among the Scandinavian samples was small to moderate with pairwise *F*_ST_ values ranging from 0.001 to 0.041 for the 82 SNPs (Table 3). Two distinct significantly differentiated genetic clusters were observed representing NW and SE Scandinavia (Table 3). An exception to this was the NW sample from Årdal (*N* = 22) that showed weak but nonsignificant differentiation (pairwise *F*_ST_ = 0.001–0.007) toward the sampling locations in the SE cluster. Within the two clusters, much smaller and nonsignificant pairwise *F*_ST_ values were observed, ranging from 0.001 to 0.009. Between samples from Scandinavia and Galicia, large significant genetic differentiation was observed, with all pairwise *F*_ST_ values ranging from 0.106 to 0.148 (Table 3). Slightly larger genetic differentiation was observed between samples within the SE Scandinavian cluster and Galicia (pairwise *F*_ST_ = 0.139–0.148), than between samples within the NW Scandinavian cluster and Galicia (pairwise *F*_ST_ = 0.106–0.144).

**TABLE 3:** Pairwise genetic differentiation (*F*_ST_) for ballan wrasse (*Labrus bergylta*) between sampling locations genotyped at 82 SNP loci, with associated *p*‐values after 10,000 permutations above diagonal. Statistically significant (alpha = 0.05) results after sequential Bonferroni correction indicated in bold. Color gradient represents relative values, with greener values indicating small differentiation (*F*_ST_ closer to zero), increasing toward red to indicate large differentiation. GalP and GalS indicate Galician Plain and Spotty phenotypes, respectively

		Northwestern (NW) Scandinavia												Southeastern (SE) Scandinavia						Spain (SP)		
		Sau	Fla	Smo	ET	Ves	Flo	Gul	Oyg	Har	Fje	Aus	Aar	Man	Flod	Ris	Hva	Str	Got	Gal	GalP	GalS
NW	Sau	*	0.712	0.567	0.269	0.675	0.760	0.083	0.224	**0.049**	**0.007**	**0.000**	0.113	**0.000**	**0.000**	**0.000**	**0.000**	**0.000**	**0.000**	**0.000**	**0.000**	**0.000**
	Fla	0.000	*	0.998	0.882	1.000	0.774	0.552	0.159	0.106	0.161	0.679	0.776	**0.000**	**0.000**	**0.000**	**0.000**	**0.000**	**0.000**	**0.000**	**0.000**	**0.000**
	Smo	0.000	0.000	*	0.503	0.493	0.352	0.208	0.285	0.239	**0.025**	0.190	0.061	**0.000**	**0.000**	**0.000**	**0.000**	**0.000**	**0.000**	**0.000**	**0.000**	**0.000**
	ET	0.002	0.000	0.000	*	0.657	0.699	0.810	0.169	0.145	0.086	**0.008**	0.189	**0.000**	**0.000**	**0.000**	**0.000**	**0.000**	**0.000**	**0.000**	**0.000**	**0.000**
	Ves	0.000	0.000	0.000	0.000	*	0.660	**0.020**	0.131	0.060	**0.002**	**0.000**	**0.047**	**0.000**	**0.000**	**0.000**	**0.000**	**0.000**	**0.000**	**0.000**	**0.000**	**0.000**
	Flo	0.000	0.000	0.001	0.000	0.000	*	0.822	0.487	0.195	0.052	**0.007**	0.073	**0.000**	**0.000**	**0.000**	**0.000**	**0.000**	**0.000**	**0.000**	**0.000**	**0.000**
	Gul	0.004	0.000	0.002	0.000	**0.005**	0.000	*	0.355	0.335	0.111	0.072	0.283	**0.000**	**0.000**	**0.000**	**0.000**	**0.000**	**0.000**	**0.000**	**0.000**	**0.000**
	Oyg	0.002	0.003	0.001	0.004	0.003	0.000	0.000	*	0.958	0.271	0.927	0.956	**0.000**	**0.000**	**0.000**	**0.000**	**0.001**	**0.000**	**0.000**	**0.000**	**0.000**
	Har	**0.004**	0.002	0.001	0.003	0.002	0.002	0.000	0.000	*	0.096	0.055	0.126	**0.000**	**0.000**	**0.000**	**0.000**	**0.000**	**0.000**	**0.000**	**0.000**	**0.000**
	Fje	**0.009**	0.002	**0.007**	0.005	**0.008**	0.006	0.004	0.002	0.003	*	0.724	0.084	**0.000**	**0.000**	**0.000**	**0.000**	**0.000**	**0.000**	**0.000**	**0.000**	**0.000**
	Aus	**0.007**	0.000	0.001	**0.006**	**0.008**	**0.006**	0.003	0.000	0.002	0.000	*	1.000	**0.000**	**0.000**	**0.000**	**0.000**	**0.000**	**0.000**	**0.000**	**0.000**	**0.000**
	Aar	0.004	0.000	0.005	0.003	**0.004**	0.005	0.001	0.000	0.003	0.005	0.000	*	**0.024**	0.089	**0.048**	**0.021**	0.225	0.287	**0.000**	**0.000**	**0.000**
	Man	**0.031**	**0.020**	**0.026**	**0.031**	**0.032**	**0.034**	**0.031**	**0.017**	**0.035**	**0.023**	**0.011**	**0.005**	*	**0.007**	**0.026**	**0.013**	0.346	0.163	**0.000**	**0.000**	**0.000**
SE	Flod	**0.035**	**0.019**	**0.025**	**0.029**	**0.034**	**0.031**	**0.029**	**0.016**	**0.031**	**0.015**	**0.012**	0.003	**0.003**	*	0.109	0.643	0.775	0.269	**0.000**	**0.000**	**0.000**
	Ris	**0.039**	**0.025**	**0.031**	**0.032**	**0.035**	**0.033**	**0.028**	**0.019**	**0.034**	**0.015**	**0.010**	**0.005**	**0.004**	0.001	*	0.740	0.653	0.051	**0.000**	**0.000**	**0.000**
	Hva	**0.038**	**0.022**	**0.029**	**0.030**	**0.035**	**0.035**	**0.030**	**0.019**	**0.036**	**0.016**	**0.011**	**0.007**	**0.004**	0.000	0.000	*	0.540	0.447	**0.000**	**0.000**	**0.000**
	Str	**0.032**	**0.019**	**0.023**	**0.025**	**0.033**	**0.029**	**0.028**	**0.012**	**0.032**	**0.016**	**0.012**	0.002	0.000	0.000	0.000	0.000	*	0.329	**0.000**	**0.000**	**0.000**
	Got	**0.041**	**0.023**	**0.031**	**0.031**	**0.038**	**0.039**	**0.033**	**0.019**	**0.035**	**0.020**	**0.012**	0.001	0.001	0.001	0.003	0.000	0.001	*	**0.000**	**0.000**	**0.000**
	Gal	**0.116**	**0.106**	**0.115**	**0.107**	**0.109**	**0.120**	**0.122**	**0.144**	**0.129**	**0.120**	**0.122**	**0.121**	**0.140**	**0.139**	**0.140**	**0.142**	**0.142**	**0.148**	*	0.475	0.343
SP	GalP	**0.110**	**0.105**	**0.110**	**0.104**	**0.104**	**0.112**	**0.119**	**0.144**	**0.125**	**0.120**	**0.121**	**0.125**	**0.146**	**0.143**	**0.149**	**0.148**	**0.146**	**0.154**	0.000	*	**0.000**
	GalS	**0.132**	**0.121**	**0.133**	**0.121**	**0.124**	**0.139**	**0.136**	**0.156**	**0.143**	**0.133**	**0.133**	**0.133**	**0.149**	**0.144**	**0.146**	**0.149**	**0.147**	**0.153**	0.001	**0.025**	*

**FIGURE 3:**
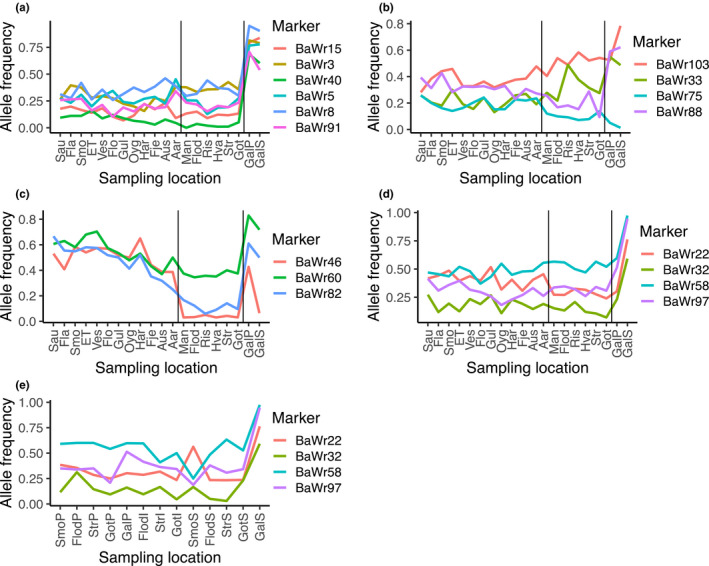
Examples of allele frequencies of SNP markers for ballan wrasse (*Labrus bergylta*) that display clear allele frequency trends: (a) between Scandinavia and Galicia, (b) with latitude, (c) for Scandinavian outlier loci, (d) for Galician outlier loci, and (e) for Galician outlier loci in all morphotype split locations (ordered by latitude and phenotype; initial uppercase letter following sampling location code indicates phenotype: P: plain; I: intermediate; and S: spotted). Black vertical lines indicate border between areas

Six SNPs showed a low or very low frequency of one allele in all samples in Scandinavia yet a high or very high frequency of the same allele in the Galician samples (Figure 3a). Several SNPs also displayed variation in allele frequencies along the north–south gradient in Scandinavia (Figure 3b). Additionally, the Scandinavian outlier loci showed major variation in allele frequencies between the two Scandinavian clusters (Figure 3c), while little variation was found for the Galician outlier loci for both Scandinavian samples and phenotypes (Figure 3d,e).

**FIGURE 4:**
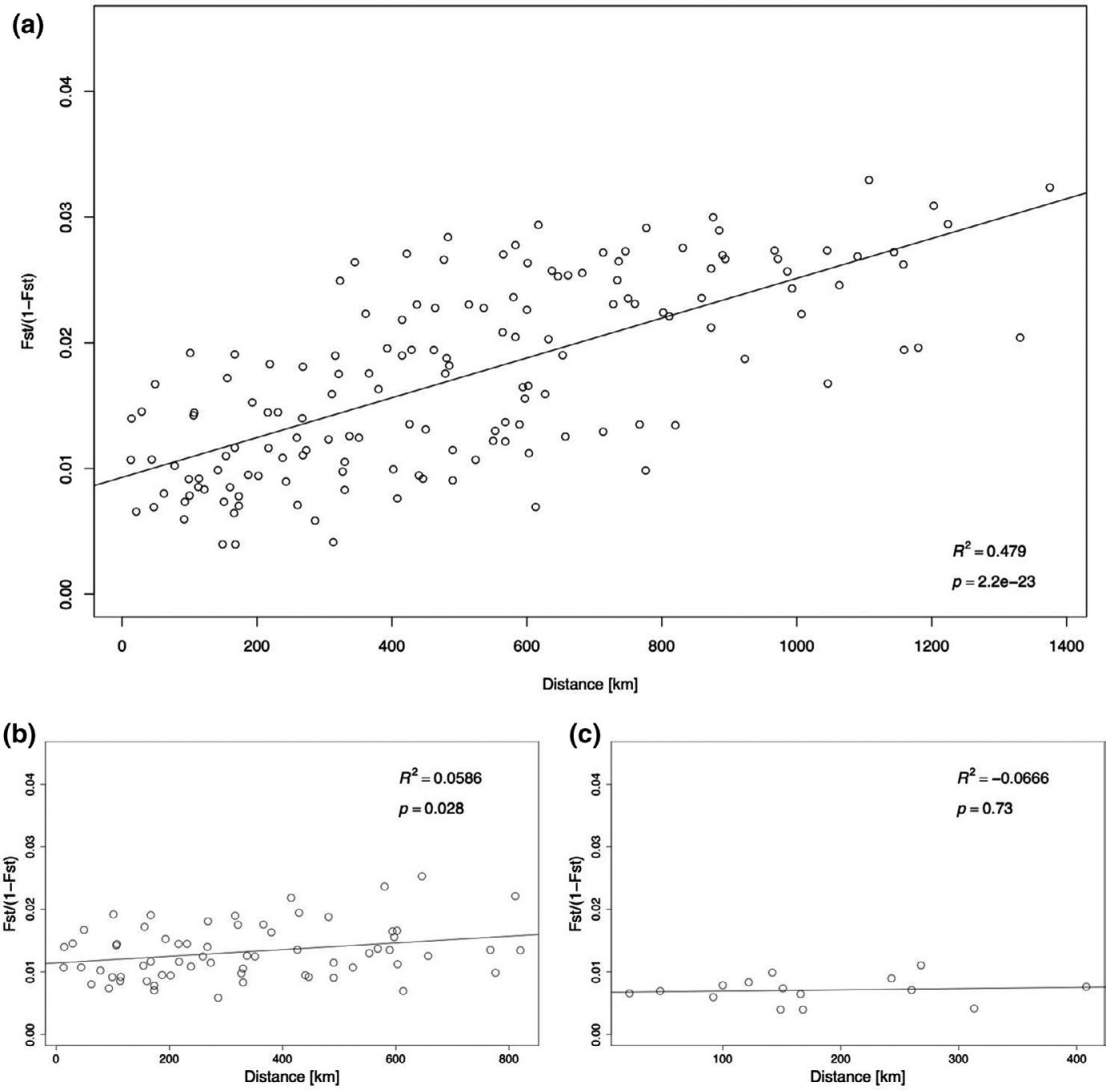
Isolation by distance (IBD) for ballan wrasse (*Labrus bergylta*) for (a) entire sampled transect from Sauhestøya to Gothenburg, (b) northwestern Scandinavia (Sauhestøya to Austevoll), and (c) southeastern Scandinavia (Mandal to Gothenburg)

Analysis of IBD showed that genetic differentiation (*F*_ST_/1−*F*_ST_) increased with increasing distance through the entire Scandinavian study area (Figure 4a). A week IBD relationship was also observed within NW (Figure 4b), while no such relationship could be found within the SE cluster (Figure 4c).

### 3.3 Clustering analysis and DAPC

**FIGURE 5:**
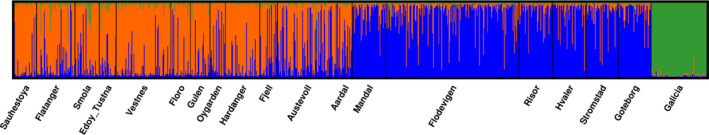
STRUCTURE bar plots (82 SNPS; *K* = 3) for ballan wrasse (*Labrus bergylta*) sampled at 19 locations in Scandinavia and Galicia, Spain. Vertical bars represent individuals, and coloration indicates assignment probability to each of the three genetic clusters. STRUCTURE analysis was also conducted without three outlier loci with no change in overall clustering (Figure S1 in Appendix S1)

Both ΔK and mean LnP(*K*) (Evanno et al., 2005) and the four alternative statistics of StructureSelector (MedMed, MedMean, MaxMed, and MaxMean; Li & Liu, 2018) indicated *K* = 3 as the most likely number of clusters within the entire dataset including samples from both Scandinavia and Galicia. When running STRUCTURE at *K* = 3, individuals were assigned to two clusters within Scandinavia and a third representing Galicia (Figure 5). Clusters within Scandinavia largely represent the NW and SE clusters also observed by pairwise *F*_ST_, with a distinct break located between Årdal and Mandal in southwestern Norway (Figure 1). Despite this clear break, some individuals from both areas (NW and SE) were assigned to the opposite genetic cluster.

**FIGURE 6:**
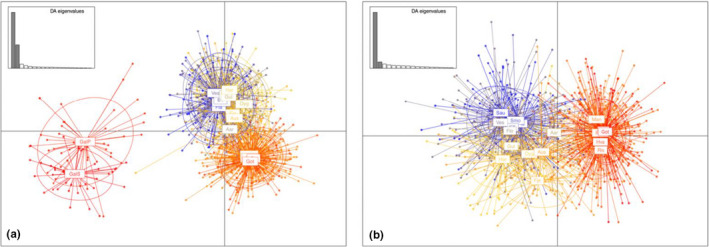
Discriminant analysis of principal components (DAPC) of ballan wrasse (*Labrus bergylta*) sampled at 18 locations in Scandinavia and in Galicia, Spain, genotyped for 82 SNPs with 60 principal components kept. Plot A showing all samples and B showing Scandinavian samples only. Projected inertia % for the axes: A: PC1 5.1% and PC2 3.0%; and B: PC1 3.4% and PC2 2.4%. Color indicates sampling location. Shading of DA eigenvalues indicates DAs kept. DAPCs were also conducted without three outlier loci with little change in clustering (Figure S3 in Appendix S1)

Results from the DAPC supported observations above (Figure 6a). The first principal component (PC1) separated Galicia and Scandinavia, while the second principal component (PC2) separated Scandinavian samples into the NW and the SE clusters (Figure 6a). The removal of the Galician samples further highlighted the differentiation between Scandinavian clusters (Figure 6b).

Both Structure and DAPC were conducted with and without the three SNPs putatively identified as under selection. The removal of these SNPs had a very minor influence on the resolution of genetic differentiation, although both of the analyses performed without these three SNPs gave slightly weaker population resolution (Figures S1 and S2).

### 3.4 Cline analysis

**FIGURE 7:**
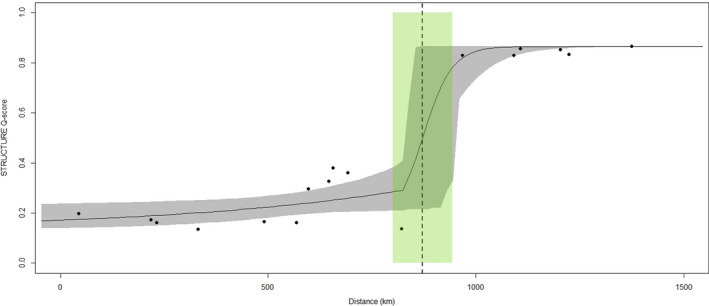
Geographic cline analysis for ballan wrasse (*Labrus bergylta*) across a 1,500‐km transect along the Scandinavian coast from Sauhestøya in Trøndelag, Norway, to Gothenburg, Sweden. Cline fitting the STRUCTURE Q‐score, with the center of the cline depicted by a vertical dashed line and the width highlighted in green

The reference cline based on the STRUCTURE Q‐score fitted a fixL model with the center situated at 874 km from Sauhestøya and showing a width of 139 km (Figure 7). This area corresponds to the Jæren coastline and surrounding areas, approximately splitting the Scandinavian dataset into NW and SE components as for all other analyses described above. Out of the 82 SNPs tested, 46 did not fit any of the 15 cline modes as their allele frequencies were stable throughout the 1,500‐km transect separating Sauhestøya and Gothenburg. In contrast, the remaining 36 SNPs showed clines with centers ranging between 295 and 1,386 km from the starting point (see Figure S3 and Table S5 for full details). Seven of the cline centers were found to overlap with the reference cline (Figure S3) with one of these markers, BaWr‐46, identified as an outlier putatively under selection within Scandinavia.

### 3.5 Genetic differentiation and Bayesian clustering of phenotype sorted samples

Moderately large and highly significant genetic differentiation was observed between the sympatric spotty and plain wrasse sampled in Galicia, as revealed by the 82 SNPs (pairwise *F*_ST_ = 0.025, *p* < .001; Table 3). This was also reflected in the DAPC (Figure 6a). In stark contrast however, no genetic differentiation was revealed among spotty, intermediate, or plain phenotypes in any of the Scandinavian samples (pairwise *F*_ST_: Smo < 0.001, *p* = .687; Flod < 0.001, *p* = .665; Str = 0.001, *p* = .453; Got = 0.013, *p* = .036; Table S6).

**FIGURE 8:**
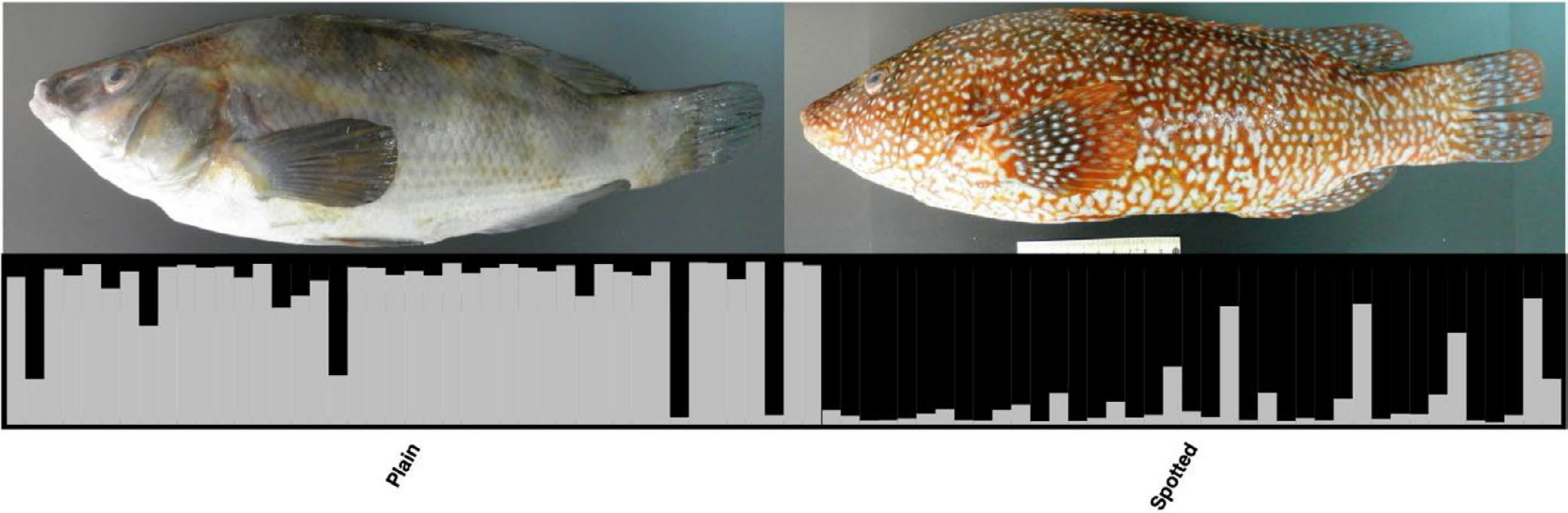
STRUCTURE bar plot (82 SNPs; *K* = 2) of Galician ballan wrasse (*Labrus bergylta*) phenotypes including example photographs of plain and spotted fish

Of the STRUCTURE runs (K = 2) for each of the phenotype sorted samples, only plain and spotty Galician phenotypes showed distinct clustering when genotyped for SNPs (Figure 8). For Scandinavian samples, no separation of phenotype was observed (Figure S4).

## 4 DISCUSSION

This study presents the most extensive analysis of population genetic structure in ballan wrasse thus far. We identified two distinct genetic clusters splitting populations in NW and SE Scandinavia and discovered very large genetic differences between all Scandinavian and Galician samples. The split in Scandinavia was located in southwestern Norway across a region that also includes sandy beaches, representing a habitat discontinuity for this species. Finally, sympatric plain and spotty phenotypes were genetically distinct in Galicia, but not in Scandinavia.

### 4.1 Genetic break in Scandinavia

A distinct break in population genetic structure was observed in Scandinavia, splitting ballan wrasse into NW and SE clusters. This break is located on the coast of southern Rogaland, in southwestern Norway, an area of coastline defined by the longest continuous stretch of sandy bottom substrate along the Norwegian coast (the Jæren beaches; ~26 km; Blanco Gonzalez, Knutsen, and Jorde (2016)). This sandy bottom would function as a habitat discontinuity for this species and is likely to be a major reason for the observed genetic break. Ballan wrasse have a strong preference for rocky habitats, and hard‐bottom substrates are necessary for them to lay their sticky benthic eggs (Darwall et al., 1992; Quignard & Pras, 1986; Villegas‐Ríos, Alós, et al., 2013). Genetic breaks in fish populations associated with discontinuities in rocky substrates by sandy areas have been described previously (e.g., Bernardi (2000), Riginos and Nachman (2001)). In particular, a similar break in the population genetic structure of the corkwing wrasse has also been observed at this exact location in Norway (Blanco Gonzalez et al., 2016; Faust et al., 2018; Mattingsdal et al., 2020), as well as in the hard‐bottom associated kelp *Laminaria hyperborea* (Evankow et al., 2019). It should be noted that for corkwing wrasse, the genetic break across the beaches of Jæren aligns with considerable differences in life‐history traits and has important implications for management (Halvorsen et al., 2016). Therefore, investigations of life‐history traits in ballan wrasse could yield a deeper understanding of the biological consequences of the observed genetic barrier.

A result that should be noted is linked to the sample from Årdal in the NW cluster. This sample was not genetically distinct from any of the other Scandinavian sampling locations. Whether this is caused by Årdal being a very small sample (i.e., by chance), close to the center of the break between the two main genetic clusters, or whether this is due to aquaculture practice is not known and cannot be determined based on the current dataset. Future studies would benefit from including multiple other samples from areas close to the genetic break, and new markers designed to distinguish between the genetic clusters identified here and potential hybridization between them.

### 4.2 Colonization of Scandinavia

The majority of the North Sea was covered by an ice sheet during the last glacial maximum (LGM; Lambeck, 1995; Shennan et al., 2000). Thus, with most northern European habitats unavailable during the LGM, species currently inhabiting northern European waters must have survived in glacial refugia. For ballan wrasse, areas along the Iberian and North African coasts could have served such a purpose as these areas upheld surface temperatures within the species thermal limits during the LGM (Almada et al., 2017; Almada, Almada, Francisco, Castilho, & Robalo, 2012). In addition, multiple possible glacial refugia have been proposed for other marine species, such as around the British Isles (Almada et al., 2012) and even in some parts of the North Sea (Gysels, Hellemans, Pampoulie, & Volckaert, 2004; Robalo et al., 2011). The observed differences in genetic variation and degree of genetic differentiation between ballan wrasse in different parts of Europe may therefore in part be caused by recolonization from different glacial refugia as proposed by Almada et al. (2017).

A decrease in mtDNA nucleotide and haplotype diversity was found by D'Arcy, Mirimin, and FitzGerald (2013) when comparing ballan wrasse from southern Norway to Great Britain (GB), and by Almada et al. (2017) when comparing ballan wrasse from southern Norway to GB and continental Europe (France and western Iberia) as well as the Azores. In the current study, all Scandinavian samples displayed significantly less genetic diversity for microsatellites in both the total number of alleles and allelic richness in comparison with the sample from Galicia. Therefore, data from previous studies and the present study collectively suggest that Scandinavian ballan wrasse populations have undergone founder effect(s) and possibly subsequent bottlenecks during the process(es) of colonization from southern latitudes. This is consistent with observations from corkwing wrasse (Knutsen et al., 2013; Robalo et al., 2011) that has a similar ecology and biology to ballan wrasse.

As ballan wrasse is a coastal species that displays a strong affiliation to rocky substrates, colonization of Scandinavia from southern latitudes would most likely have followed the coastline northward. This suggests that colonization could have followed the coastline of mainland Europe to Scandinavia and northward, or alternatively, via the British Isles and across the North Sea to western Norway and thereafter both northward and southward in Scandinavia. The latter has been proposed as the main route of colonization of Scandinavia for the corkwing wrasse (Knutsen et al., 2013; Mattingsdal et al., 2020). Our data also suggest this to be most likely for ballan wrasse as we observed greater genetic differentiation between SE Scandinavia and Galicia than between NW Scandinavia and Galicia. This also fits better with climatological records which show that western Norway was probably available prior to Skagerrak and other areas in eastern Scandinavia (Stroeven et al., 2016), and geological records, showing that the English channel and large areas of the North Sea was dry land during and immediately following the LGM (Lambeck, 1995; Shennan et al., 2000). In order to clarify this, future studies of ballan wrasse need to encompass samples from the entire distribution range, especially the British Isles and Denmark to Atlantic France.

### 4.3 Phenotypic and genetic variation

In Galicia, sympatric spotty and plain phenotypes have been shown to display overlapping yet differing life‐history strategies, with plain fish investing more in reproduction while spotty fish invest more in growth (Villegas‐Ríos, Alonso‐Fernández, Fabeiro, et al., 2013). These observations indicate that the two phenotypes represent different genetic groups that have even been suggested to be potentially different subspecies (Quintela et al., 2016; Villegas‐Ríos, 2013). Quintela et al. (2016) reported a weak but significant genetic differentiation between sympatric plain and spotty phenotypes of ballan wrasse in Galicia using microsatellites. The results of our study have expanded upon this previous work by analyzing phenotyped samples from multiple locations with a panel of SNPs developed here. For the Galician samples, the 82 SNPs revealed clear genetic differentiation between both phenotypes (*F*_ST_ = 0.025; Table 3). As such, the present study provides the most compelling evidence yet that in the southern part of its distribution range, spotty and plain ballan wrasse represent distinct groups. However, and in stark contrast, no genetic differentiation was detected between the sympatric spotty and plain phenotypes in any of the four Scandinavian samples where this was investigated (see Table 1 for locations).

Previous authors have suggested that the two sympatric phenotypes in Galicia could be maintained through assortative mating (Villegas‐Ríos, Alonso‐Fernández, Domínguez‐Petit, et al., 2013). It is also possible that other mechanisms are at play; for example, the two phenotypes may spawn at subtly different times or areas or that the two phenotypes have selective advantages in different parts of the environment. Whatever the underlying mechanisms, a significant question is why they are at play in the southern range of this species, but not in Scandinavia? Spot patterns in fish may be both environmentally and genetically determined (Jørgensen et al., 2018; Kause, Ritola, Paananen, Eskelinen, & Mantysaari, 2003; Skaala & Jørstad, 1987), and as such, differences in abiotic factors may result in less spotty fish in northern latitudes. Furthermore, although the two phenotypes are present in Scandinavia (Villegas‐Ríos, Alonso‐Fernández, Fabeiro, et al., 2013), phenotypic variation was observed to be less distinct than in Galicia, with a third intermediate category also present. Therefore, it is theoretically possible that differences in environmental conditions between Scandinavia and Galicia may subtly modify spotting patterns or other trait characteristics of these two phenotypes in the southern latitudes, so that the mechanisms holding them distinct in the south are not at play in Scandinavia. Accordingly, if the two phenotypes are somehow maintained through a selective advantage to a habitat in Galicia that is not present in Scandinavia, the differentiation between the phenotypic groups and associated genetic groups would collapse. Further studies using reciprocal crossing experiments under different environmental conditions, as has been done in Atlantic salmon (Jørgensen et al., 2018), could be used to disentangle this issue.

### 4.4 Management implications

The management of wrasse in Scandinavia is currently facing two major challenges, both linked to their extensive use as cleaner fish to delouse farmed salmonids in sea‐cages. These challenges include the sustainable harvest of wild populations to provide cleaner fish for the aquaculture industry, and the potential aquaculture‐driven inadvertent translocation of wrasse from southern Norway and Sweden to western and middle Norway. Results from the present study are highly relevant in order to address both these issues.

We observed a distinct genetic difference between ballan wrasse in SE and NW Scandinavia. Therefore, our data clearly demonstrate that the inadvertent translocation of wrasse, via current aquaculture practice, from Sweden and southern Norway to western and middle Norway, will result in mixing fish from highly distinct genetic groups (and potentially phylogenetic lineages). Therefore, this aquaculture practice requires revision in light of our data. Studies of other species of wrasse subjected to the same aquaculture‐driven harvest and translocation regimes have indicated escapes and potential hybridization between translocated individuals and local populations (Faust et al., 2018; Jansson et al., 2017). However, while the genetic markers implemented here provided a clear picture of population genetic structure between NW and SE Scandinavia, they do not provide enough statistical power to accurately identify potential escaped fish or hybrids sampled in the middle part of Norway. In corkwing wrasse, several thousand SNPs were screened to develop population‐diagnostic loci that could accurately identify escapees, hybrids, and backcrosses (Faust et al., 2018). We therefore suggest that a more comprehensive set of genetic markers is required to investigate the degree to which translocated ballan wrasse survive and hybridize with local wild populations.

Harvest of wild wrasses has dramatically risen in Norway and Sweden (Norwegian Directorate of Fisheries, n.d.; Rueness et al., 2019) in order to meet the aquaculture industry's demand for cleaner fish. This sudden increase in exploitation of a previously noncommercial species raises the question of sustainability and potential extinction of local populations (Halvorsen et al., 2017). Failure to take the spatial and temporal extent of populations into account when harvesting living resources can lead to differential exploitation and potentially overexploitation (Allendorf, England, Luikart, Ritchie, & Ryman, 2008; Kerr et al., 2017). The concern that labrids, and especially ballan wrasse, are disappearing from multiple areas in Norway have already been raised both by the public and by fishermen that report having to move to new fishing locations as catches decline (Grefsrud et al., 2018). This suggests that ballan wrasse are very stationary, which has also been shown in studies of site fidelity in Galicia (Mucientes et al., 2019). The data presented here show some evidence of genetic structuring within the SE and NW Scandinavian clusters. This needs further investigation, combining biological, fishery and genetic data, in order to better understand connectivity within regions, and thus the potential for exhaustion of local populations.

In Galicia, the two phenotypic groups are already sold and marketed separately (Villegas‐Ríos, Alonso‐Fernández, Fabeiro, et al., 2013) and are therefore likely subject to different fishing pressures. The findings of this study could thus be used as a scientific basis for upholding this practice, and as a basis for the future management of these two groups as separate stocks.

## CONFLICT OF INTEREST

None declared.

## AUTHOR CONTRIBUTIONS

**Gaute W. Seljestad:** Data curation (lead); Formal analysis (lead); Investigation (lead); Visualization (lead); Writing‐original draft (lead); Writing‐review & editing (lead). **María Quintela:** Formal analysis (supporting); Resources (supporting); Supervision (supporting); Writing‐review & editing (supporting). **Ellika Faust:** Resources (supporting); Writing‐review & editing (supporting). **Kim T. Halvorsen:** Resources (supporting); Writing‐review & editing (supporting). **François Besnier:** Formal analysis (supporting); Writing‐review & editing (supporting). **Eeva Jansson:** Investigation (supporting); Resources (supporting); Writing‐review & editing (supporting). **Geir Dahle:** Data curation (supporting); Formal analysis (supporting); Writing‐review & editing (supporting). **Halvor Knutsen:** Funding acquisition (supporting); Writing‐review & editing (supporting). **Carl André:** Funding acquisition (supporting); Resources (supporting); Writing‐review & editing (supporting). **Arild Folkvord:** Supervision (supporting); Writing‐review & editing (supporting). **Kevin A. Glover:** Conceptualization (lead); Project administration (lead); Supervision (lead); Writing‐original draft (supporting); Writing‐review & editing (supporting).

## Supporting information

Appendix S1

## Data Availability

The raw data underlying the main results for this study are archived and available in Dryad at https://doi.org/10.5061/dryad.3n5tb2rdd.
