# Supplementary material for: “A cleaner break”: Genetic divergence between geographic groups and sympatric phenotypes revealed in ballan wrasse (Labrus bergylta)
Source: Ecol Evol. 2020 Jun 5;10(12):6120–35. doi: 10.1002/ece3.6404 (PMC7319121; doi:10.1002/ece3.6404)
Supplement: Supplementary file 1 — Appendix S1 [file ECE3-10-6120-s001.docx]

# Appendix: Additional information and results

| **Table A1** Overview of ballan wrasse multiplex (MP) 1 including volumes of PCR Primers and Unextended Extend Primers (UEP). Markers in yellow removed from multiplex before this study was initiated. | | | | |
| --- | --- | --- | --- | --- |
| **Multiplex** | **SNP_ID** | **Marker name** | **PCR Primers (1st & 2nd)** | **UEP** |
|  |  |  | **Total volume** | **Total volume** |
|  |  |  | **1000** | **1500** |
| MP1 | NW_018114517.1_953305 | BaWr-1 | 5 | 21 |
| MP1 | NW_018114501.1_1110519 | BaWr-2 |  |  |
| MP1 | NW_018114781.1_75751 | BaWr-3 | 5 | 21 |
| MP1 | NW_018114604.1_365777 | BaWr-4 | 5 | 21 |
| MP1 | NW_018114505.1_499976 | BaWr-5 | 5 | 21 |
| MP1 | NW_018114434.1_45754 | BaWr-6 | 5 | 21 |
| MP1 | NW_018114474.1_1618630 | BaWr-7 | 5 | 21 |
| MP1 | NW_018114489.1_483994 | BaWr-8 | 5 | 21 |
| MP1 | NW_018114526.1_227777 | BaWr-9 | 5 | 28 |
| MP1 | NW_018115046.1_126119 | BaWr-10 | 5 | 28 |
| MP1 | NW_018114560.1_1014493 | BaWr-11 | 5 | 28 |
| MP1 | NW_018114681.1_636455 | BaWr-12 | 5 | 28 |
| MP1 | NW_018114571.1_854545 | BaWr-13 | 5 | 28 |
| MP1 | NW_018114696.1_226866 | BaWr-14 | 5 | 28 |
| MP1 | NW_018114523.1_1049816 | BaWr-15 | 5 | 35 |
| MP1 | NW_018114483.1_1338510 | BaWr-16 |  |  |
| MP1 | NW_018114481.1_912790 | BaWr-17 | 5 | 35 |
| MP1 | NW_018114757.1_45148 | BaWr-18 | 5 | 35 |
| MP1 | NW_018114486.1_1620397 | BaWr-19 | 5 | 35 |
| MP1 | NW_018114444.1_1460970 | BaWr-20 | 5 | 35 |
| MP1 | NW_018114436.1_1764087 | BaWr-21 | 5 | 35 |
| MP1 | NW_018114461.1_1600142 | BaWr-22 | 5 | 42 |
| MP1 | NW_018114430.1_251585 | BaWr-23 | 5 | 42 |
| MP1 | NW_018114471.1_453074 | BaWr-24 |  |  |
| MP1 | NW_018114451.1_1926182 | BaWr-25 | 5 | 42 |
| MP1 | NW_018114428.1_906974 | BaWr-26 | 5 | 42 |
| MP1 | NW_018114714.1_15927 | BaWr-27 |  |  |
| MP1 | NW_018114433.1_2198459 | BaWr-28 | 5 | 42 |
| MP1 | NW_018114500.1_827265 | BaWr-29 | 5 | 42 |
| **Combined primer volume** | | | **750** | **723** |

| **Table A2** Overview of ballan wrasse multiplex (MP) 2 including volumes of PCR Primers and Unextended Extend Primers (UEP). Markers in yellow removed from multiplex before this study was initiated. | | | | |
| --- | --- | --- | --- | --- |
| **Multiplex** | **SNP_ID** | **Marker name** | **PCR Primers (1st & 2nd)** | **UEP** |
|  |  |  | **Total volume** | **Total volume** |
|  |  |  | **1000** | **1500** |
| MP2 | NW_018114480.1_686536 | BaWr-30 | 5 | 21 |
| MP2 | NW_018114648.1_639468 | BaWr-31 | 5 | 21 |
| MP2 | NW_018114431.1_1403930 | BaWr-32 | 5 | 21 |
| MP2 | NW_018114885.1_120757 | BaWr-33 | 5 | 21 |
| MP2 | NW_018114418.1_2113518 | BaWr-34 | 5 | 21 |
| MP2 | NW_018114458.1_853662 | BaWr-35 | 5 | 21 |
| MP2 | NW_018114580.1_402395 | BaWr-36 | 5 | 28 |
| MP2 | NW_018114519.1_401347 | BaWr-37 | 5 | 28 |
| MP2 | NW_018114435.1_1416097 | BaWr-38 |  |  |
| MP2 | NW_018114475.1_1475041 | BaWr-39 | 5 | 28 |
| MP2 | NW_018114728.1_554763 | BaWr-40 | 5 | 28 |
| MP2 | NW_018114480.1_1226704 | BaWr-41 | 5 | 28 |
| MP2 | NW_018114455.1_84460 | BaWr-42 | 5 | 28 |
| MP2 | NW_018114465.1_196310 | BaWr-43 | 5 | 28 |
| MP2 | NW_018114441.1_653994 | BaWr-44 | 5 | 35 |
| MP2 | NW_018114447.1_1776371 | BaWr-45 |  |  |
| MP2 | NW_018114416.1_56373 | BaWr-46 | 5 | 35 |
| MP2 | NW_018114511.1_656939 | BaWr-47 | 5 | 35 |
| MP2 | NW_018114487.1_1240633 | BaWr-48 | 5 | 35 |
| MP2 | NW_018114532.1_341495 | BaWr-49 | 5 | 35 |
| MP2 | NW_018114593.1_501629 | BaWr-50 | 5 | 42 |
| MP2 | NW_018114492.1_409355 | BaWr-51 |  |  |
| MP2 | NW_018114468.1_189838 | BaWr-52 | 5 | 42 |
| MP2 | NW_018114449.1_1926310 | BaWr-53 | 5 | 42 |
| MP2 | NW_018114496.1_554438 | BaWr-54 | 5 | 42 |
| MP2 | NW_018114520.1_353049 | BaWr-55 | 5 | 42 |
| MP2 | NW_018114493.1_270364 | BaWr-56 |  |  |
| **Combined primer volume** | | | **770** | **786** |

| **Table A3** Overview of ballan wrasse multiplex (MP) 3 including volumes of PCR Primers and Unextended Extend Primers (UEP). Marker in yellow removed from multiplex before this study was initiated. | | | | |
| --- | --- | --- | --- | --- |
| **Multiplex** | **SNP_ID** | **Marker name** | **PCR Primers (1st & 2nd)** | **UEP** |
|  |  |  | **Total volume** | **Total volume** |
|  |  |  | **1000** | **1500** |
| MP3 | NW_018114442.1_1221044 | BaWr-57 | 5 | 21 |
| MP3 | NW_018114498.1_748966 | BaWr-58 | 5 | 21 |
| MP3 | NW_018114546.1_746859 | BaWr-59 | 5 | 21 |
| MP3 | NW_018114587.1_851110 | BaWr-60 | 5 | 21 |
| MP3 | NW_018114552.1_430323 | BaWr-61 | 5 | 21 |
| MP3 | NW_018114454.1_1274351 | BaWr-62 | 5 | 21 |
| MP3 | NW_018114482.1_1351282 | BaWr-63 | 5 | 21 |
| MP3 | NW_018115568.1_16432 | BaWr-64 | 5 | 28 |
| MP3 | NW_018114443.1_78542 | BaWr-65 | 5 | 28 |
| MP3 | NW_018114425.1_2899410 | BaWr-66 | 5 | 28 |
| MP3 | NW_018114525.1_1112059 | BaWr-67 | 5 | 28 |
| MP3 | NW_018114614.1_940723 | BaWr-68 | 5 | 28 |
| MP3 | NW_018114499.1_643624 | BaWr-69 | 5 | 28 |
| MP3 | NW_018114452.1_575674 | BaWr-70 | 5 | 35 |
| MP3 | NW_018114476.1_1062158 | BaWr-71 |  |  |
| MP3 | NW_018115024.1_46771 | BaWr-72 | 5 | 35 |
| MP3 | NW_018114508.1_1255457 | BaWr-73 | 5 | 35 |
| MP3 | NW_018114420.1_1919501 | BaWr-74 | 5 | 35 |
| MP3 | NW_018114802.1_440534 | BaWr-75 | 5 | 35 |
| MP3 | NW_018114462.1_1987249 | BaWr-76 | 5 | 35 |
| MP3 | NW_018114439.1_366350 | BaWr-77 | 5 | 42 |
| MP3 | NW_018114453.1_383341 | BaWr-78 | 5 | 42 |
| MP3 | NW_018114491.1_295879 | BaWr-79 | 5 | 42 |
| MP3 | NW_018114662.1_372392 | BaWr-80 | 5 | 42 |
| MP3 | NW_018114669.1_516151 | BaWr-81 | 5 | 42 |
| MP3 | NW_018114565.1_470130 | BaWr-82 | 5 | 42 |
| **Combined primer volume** | | | **750** | **723** |

| **Table A4** Overview of ballan wrasse multiplex (MP) 4 including volumes of PCR Primers and Unextended Extend Primers (UEP). Marker in yellow removed from multiplex before this study was initiated. | | | | |
| --- | --- | --- | --- | --- |
| **Multiplex** | **SNP_ID** | **Marker name** | **PCR Primers (1st & 2nd)** | **UEP** |
|  |  |  | **Total volume** | **Total volume** |
|  |  |  | **1000** | **1500** |
| W4 | NW_018114950.1_24492 | BaWr-83 | 5 | 21 |
| W4 | NW_018114537.1_993619 | BaWr-84 | 5 | 21 |
| W4 | NW_018114575.1_206829 | BaWr-85 | 5 | 21 |
| W4 | NW_018114427.1_1443862 | BaWr-86 | 5 | 21 |
| W4 | NW_018114527.1_705419 | BaWr-87 | 5 | 21 |
| W4 | NW_018114494.1_1254704 | BaWr-88 | 5 | 21 |
| W4 | NW_018114426.1_930380 | BaWr-89 | 5 | 28 |
| W4 | NW_018114485.1_242312 | BaWr-90 | 5 | 28 |
| W4 | NW_018114522.1_178152 | BaWr-91 | 5 | 28 |
| W4 | NW_018114466.1_573987 | BaWr-92 | 5 | 28 |
| W4 | NW_018115213.1_20609 | BaWr-93 | 5 | 28 |
| W4 | NW_018115108.1_157252 | BaWr-94 | 5 | 28 |
| W4 | NW_018114557.1_105885 | BaWr-95 | 5 | 35 |
| W4 | NW_018114470.1_1952717 | BaWr-96 | 5 | 35 |
| W4 | NW_018114424.1_1723121 | BaWr-97 | 5 | 35 |
| W4 | NW_018114472.1_763740 | BaWr-98 | 5 | 35 |
| W4 | NW_018114627.1_575991 | BaWr-99 | 5 | 35 |
| W4 | NW_018114528.1_354537 | BaWr-100 | 5 | 35 |
| W4 | NW_018114437.1_23329 | BaWr-101 | 5 | 42 |
| W4 | NW_018114464.1_749310 | BaWr-102 | 5 | 42 |
| W4 | NW_018114417.1_2346164 | BaWr-103 | 5 | 42 |
| W4 | NW_018114432.1_2621672 | BaWr-104 | 5 | 42 |
| W4 | NW_018114457.1_1317312 | BaWr-105 | 5 | 42 |
| W4 | NW_018114484.1_155855 | BaWr-106 |  |  |
| **Combined primer volume** | | | **770** | **786** |

| **Table A5** Model-fitting for the different markers and parameter estimates for the geographic cline ranging from Sauhestøya to Gothenburg. Markers with clines overlapping in width with the reference Q-score are depicted in boldface type. The Table does not include the 46 loci with allele frequency not meeting cline conditions.^[[1]](#footnote-1)^ | | | | | | | | | | |
| --- | --- | --- | --- | --- | --- | --- | --- | --- | --- | --- |
| **Marker** | **Model** | **Centre (km)** | **Width (km)** | **δ_L_** | **τ_L_** | **δ_M_** | **τ_M_** | **pmin** | **pmax** | **loglike** |
| STRUCTURE Q | fixL | 874.4 | 138.7 | 45.2495 | 0.0733 | NA | NA | 0.1350 | 0.8650 | -11.3274 |
| PCA_82 SNPs | typM | 947.5 | 71.2 | NA | NA | 18.5849 | 0.0322 | 0.0000 | 1.0000 | -32.8957 |
| BaWr4 | fixN | 615.8 | 2487.8 | NA | NA | NA | NA | 0.1880 | 0.3790 | -3.2808 |
| BaWr6 | fixN | 517.7 | 1676.4 | NA | NA | NA | NA | 0.3850 | 0.6000 | -3.7905 |
| BaWr11 | fixN | 784.8 | 2888.4 | NA | NA | NA | NA | 0.6720 | 0.8650 | -2.7914 |
| BaWr20 | fixN | 652.8 | 2262.0 | NA | NA | NA | NA | 0.5000 | 0.6880 | -2.9272 |
| BaWr22 | fixN | 676.0 | 1192.1 | NA | NA | NA | NA | 0.2400 | 0.5170 | -3.4777 |
| **BaWr28** | optN | 941.7 | 12.6 | NA | NA | NA | NA | 0.3565 | 0.5258 | -5.9477 |
| BaWr29 | fixN | 758.3 | 3597.4 | NA | NA | NA | NA | 0.2860 | 0.6420 | -7.7079 |
| BaWr32 | fixN | 629.2 | 3138.8 | NA | NA | NA | NA | 0.7320 | 0.9290 | -6.4987 |
| BaWr33 | optN | 1105.3 | 7.7 | NA | NA | NA | NA | 0.2265 | 0.3600 | -6.8513 |
| BaWr34 | fixN | 1314.5 | 3597.1 | NA | NA | NA | NA | 0.3890 | 0.7250 | -6.5006 |
| BaWr36 | fixN | 737.4 | 2739.8 | NA | NA | NA | NA | 0.8130 | 0.9580 | -4.1455 |
| BaWr40 | fixN | 449.2 | 1721.0 | NA | NA | NA | NA | 0.0000 | 0.1600 | -5.4941 |
| **BaWr46** | optN | 868.3 | 13.4 | NA | NA | NA | NA | 0.4978 | 0.9652 | -8.3079 |
| **BaWr48** | optN | 897.1 | 14.2 | NA | NA | NA | NA | 0.4684 | 0.5722 | -3.9052 |
| **BaWr54** | fixN | 848.6 | 1291.0 | NA | NA | NA | NA | 0.1190 | 0.2550 | -1.3241 |
| BaWr55 | optN | 955.8 | 0.1 | NA | NA | NA | NA | 0.6553 | 0.7833 | -3.0977 |
| BaWr58 | fixN | 319.1 | 2267.4 | NA | NA | NA | NA | 0.3700 | 0.5650 | -2.9698 |
| BaWr60 | fixN | 567.7 | 672.3 | NA | NA | NA | NA | 0.3450 | 0.7040 | -4.7240 |
| BaWr62 | fixN | 726.5 | 1841.5 | NA | NA | NA | NA | 0.8670 | 0.9830 | -3.3752 |
| BaWr63 | fixN | 686.9 | 3221.5 | NA | NA | NA | NA | 0.2500 | 0.5100 | -4.0062 |
| BaWr72 | fixN | 519.1 | 1416.2 | NA | NA | NA | NA | 0.3330 | 0.5430 | -3.6004 |
| **BaWr75** | optN | 880.1 | 238.1 | NA | NA | NA | NA | 0.8076 | 0.9056 | -2.1963 |
| BaWr76 | fixN | 449.7 | 2425.0 | NA | NA | NA | NA | 0.8240 | 0.9780 | -4.2932 |
| BaWr78 | fixN | 532.3 | 1511.8 | NA | NA | NA | NA | 0.2130 | 0.5420 | -3.8121 |
| BaWr82 | fixN | 781.4 | 678.8 | NA | NA | NA | NA | 0.0600 | 0.5800 | -9.9821 |
| BaWr85 | fixN | 1008.3 | 2853.4 | NA | NA | NA | NA | 0.5170 | 0.7860 | -5.7847 |
| **BaWr86** | fixN | 922.1 | 1390.7 | NA | NA | NA | NA | 0.1170 | 0.3250 | -3.9833 |
| BaWr87 | fixN | 981.9 | 1510.1 | NA | NA | NA | NA | 0.8230 | 0.9550 | -2.8313 |
| BaWr88 | fixN | 773.0 | 1596.5 | NA | NA | NA | NA | 0.5740 | 0.9060 | -5.8110 |
| BaWr90 | fixN | 691.6 | 1966.8 | NA | NA | NA | NA | 0.6030 | 0.7500 | -3.5275 |
| BaWr93 | fixN | 295.4 | 2706.7 | NA | NA | NA | NA | 0.3650 | 0.6220 | -3.1814 |
| BaWr99 | fixN | 802.7 | 1452.9 | NA | NA | NA | NA | 0.7870 | 0.9040 | -3.0169 |
| **BaWr101** | fixN | 939.2 | 2532.4 | NA | NA | NA | NA | 0.1190 | 0.3020 | -3.3098 |
| BaWr103 | optN | 988.1 | 53.4 | NA | NA | NA | NA | 0.3709 | 0.5355 | -2.3716 |
| BaWr104 | fixN | 1386.3 | 3591.5 | NA | NA | NA | NA | 0.3790 | 0.7000 | -4.5359 |
| BaWr105 | fixN | 1006.6 | 3593.8 | NA | NA | NA | NA | 0.2000 | 0.5670 | -4.6073 |

| **Table A6** Pairwise genetic differentiation (*F*_ST_) between phenotype sorted ballan wrasse (*Labrus bergylta*) samples from northwestern (NW) and southeastern (SE) Scandinavia, as well as one sample from Galicia, Spain. Results statistically significant after sequential Bonferroni corrections indicated in bold. | | | |
| --- | --- | --- | --- |
| **Area** | **Sampling location** | **Pairwise *F*_ST_** | **p-value** |
| **NW** | **Smøla** |  |  |
|  | Plain vs. spotty | 0.000 | 0.687 |
| **SE** | **Flødevigen** |  |  |
|  | Plain vs. spotty | 0.000 | 0.665 |
|  | Plain vs. intermediate | 0.000 | 0.479 |
|  | Spotty vs. intermediate | 0.005 | 0.048 |
|  | **Strømstad** |  |  |
|  | Plain vs. spotty | 0.001 | 0.453 |
|  | Plain vs. intermediate | 0.000 | 0.706 |
|  | Spotty vs. intermediate | 0.000 | 0.710 |
|  | **Gothenburg** |  |  |
|  | Plain vs. spotty | 0.013 | 0.036 |
|  | Plain vs. intermediate | 0.008 | 0.112 |
|  | Spotty vs. intermediate | 0.002 | 0.377 |
| **Spain** | **Galicia** |  |  |
|  | Plain vs. spotty | **0.031** | **0.000** |


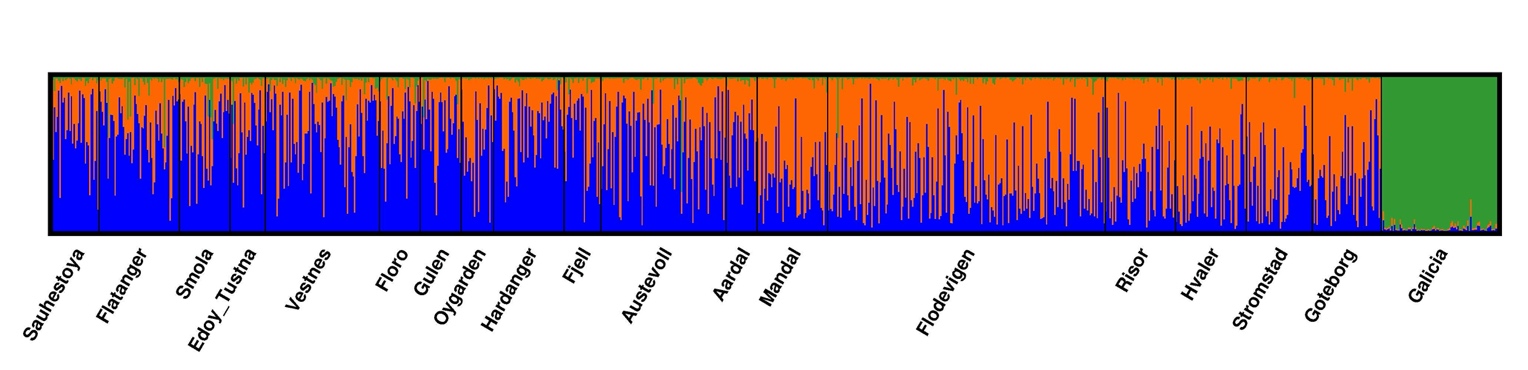


**Figure A1** STRUCTURE bar plots for ballan wrasse (*Labrus bergylta*) sampled at 19 locations at K = 3 for 79 SNPs after removal of 3 loci under directional selection. Each vertical line represents one individual, and colors indicate genetic clusters (i.e., groups). Majority of individuals in north-western Norway being assigned mostly to cluster 1, while south-eastern individuals are assigned to cluster 2, and Galician individuals making up cluster 3.

***
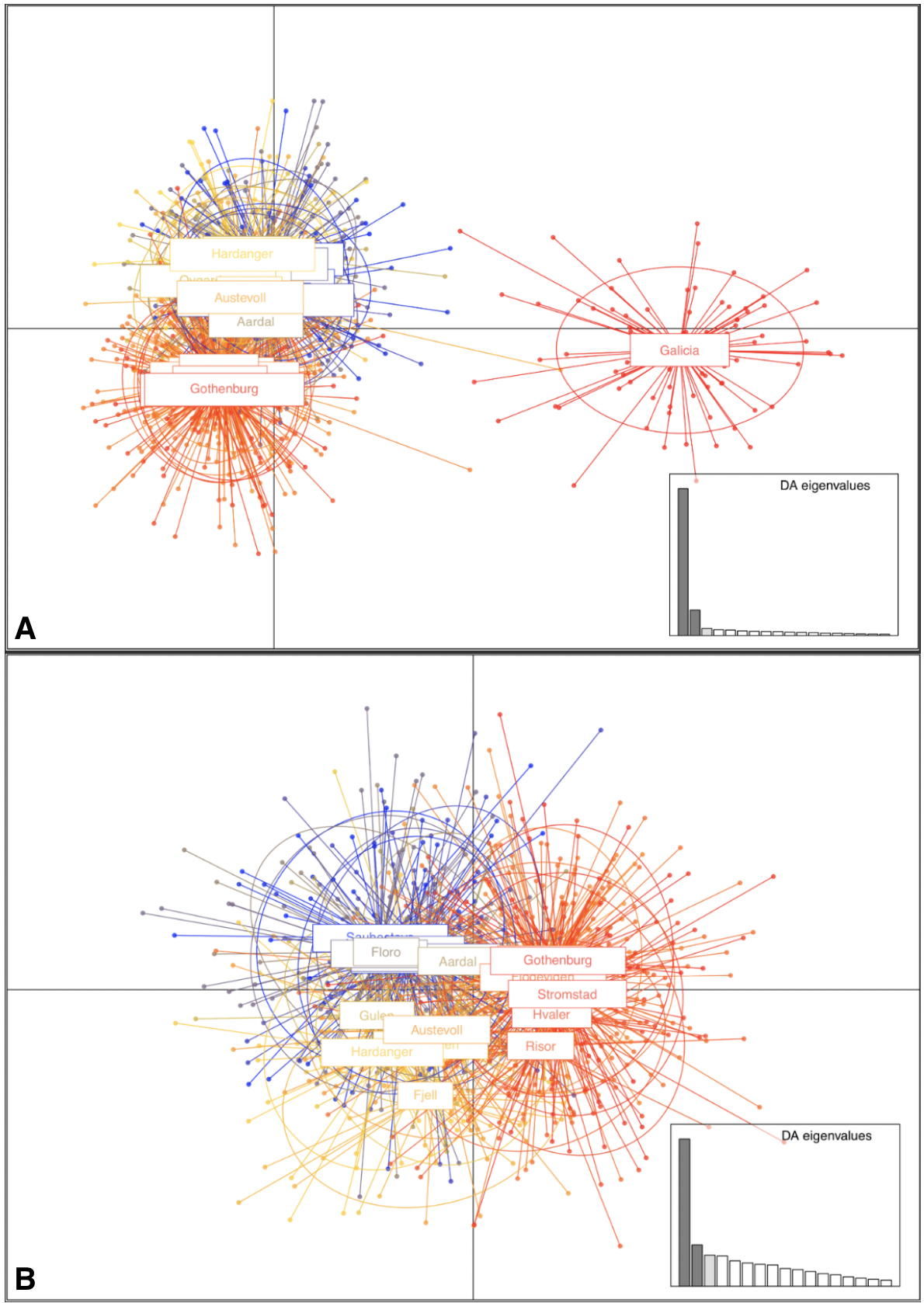
***

**Figure A2** Discriminant analysis of principle components (DAPC) for ballan wrasse (*Labrus bergylta*) sampled at 19 locations, without three loci putatively under selection, of a) all 19 locations (incl. Galicia), and b) all Scandinavian locations. Based on 79 SNPs. Coloration based on sampling location. Shading of DA eigenvalues signifies Discriminant analysis kept in the analysis.

**Figure A3** Compilation of the position of genetic clines of SNP markers: Centre and the corresponding two log-likelihood unit support limits for SNPs that showed variation with latitude. STRUCTURE reference cline is depicted by dotted red lines.


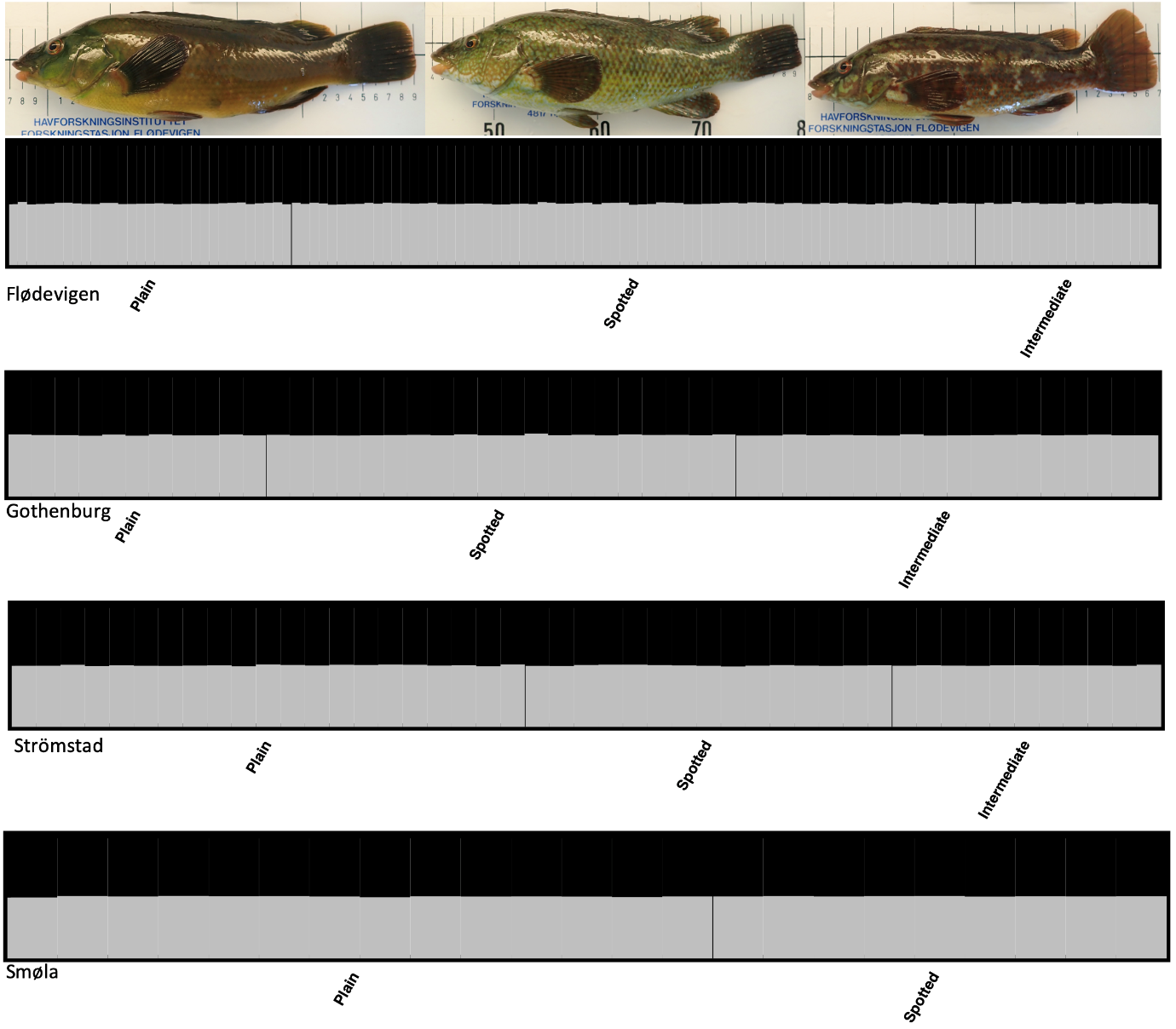


**Figure A4** STRUCTURE analysis (K = 2) of Scandinavian ballan wrasse (*Labrus bergylta*) sorted by phenotype and genotyped for 82 SNPs. Example photos of Scandinavian plain, spotted, and intermediate morphotype fish sampled in Flødevigen, Norway.

1. For the given models p_min_ and p_max_ were fixed to 0 and 1 (typ models). or to their empirical values (fix models). or p_min_ and p_max_ are fitted (opt model). Tail fitting encompassed right (R). left (L). none (N) or both fitted (B). The cline width (w) was calculated as 1/maximum slope. Two log-likelihood unit support limits are presented in parentheses for centre and width. Δ and τ are the shape parameters for the left and right tails. and p_min_ and p_max_ are the character states at either end of the transect. [↑](#footnote-ref-1)
